# Effect of Mitomycin - C and Triamcinolone on Preventing Urethral Strictures

**DOI:** 10.1590/S1677-5538.IBJU.2016.0191

**Published:** 2017

**Authors:** Omer Kurt, Fethullah Gevher, Cenk Murat Yazici, Mustafa Erboga, Mucahit Dogru, Cevat Aktas

**Affiliations:** 1 Department of Urology, Namık Kemal University, School of Medicine, Tekirdag, Turkey; 2 Department of Urology, Anadolu Hospital, Istanbul, Turkey; 3 Department of Histology, Namık Kemal University, School of Medicine, Tekirdag, Turkey; 4 Department of Radiology, Namık Kemal University, School of Medicine, Tekirdag, Turkey

**Keywords:** Endoscopy, Mitomycin, Triamcinolone, Urethral Stricture

## Abstract

Urethral stricture is a common disease with high recurrence rate. Several manipulations were defined to prevent the recurrence but the results were disappointing. This study aimed to evaluate the efficacy of triamcinolone and mitomycin-C on urethral stricture formation and their effect on inhibition of urethral fibrosis. A total of 24 New Zealand rabbits were divided into 3 groups. Urethras of rabbits were traumatized with pediatric resectoscope. Resection area was irrigated with 10mL saline, swapped with a cotton wool soaked with 0.5mg/mL MMC and injected by 40mg triamcinolone in groups 1, 2 and 3 respectively. Retrograde urethrogram was performed at 28^th^ day of procedure and the urethra was removed for histopathologic evaluation. There were significant differences in urethral diameters and in lumen reduction rate between the control and study groups (p<0.001). Compared to control group, all treatment groups showed mild fibrosis, less collagen bundle irregularity, and lower numbers of fibroblasts (p=0.003). The Tunnel assay showed that the number of apoptotic cells in the submucosal connective tissue was quantitatively higher in control groups (p=0.034). In the view of efficacy and safety, MMC and triamcinolone have the potential to replace the use of stents, clean intermittent catheterization, or long term catheters following internal urethrotomy. There were no statistically significant differences between two agents in terms of preventing urethral stricture formation in the present study. Mitomycin C and triamcinolone decreased the recurrence rates of urethral stricture.

## INTRODUCTION

Urethral stricture is one of the oldest known urologic diseases and remains a common problem with high morbidity. Injuries to the urethral epithelium or the underlying corpus spongiosum may result in scar formation leading to urethral stricture which negatively affects quality of voiding (1). Although it can occur anywhere in the urethra, about half of cases are present in the bulbar urethra. The most common causes of urethral stricture are idiopathic causes, iatrogenic causes (catheterization and transurethral surgery), inflammatory causes and trauma (pelvic fracture) (2).

Urethral stricture has been mostly treated with urethral dilation and visual internal urethrotomy (DVIU). Long-term success rate of endoscopic treatments, however, is not high. Santucci et al. showed that the long term success rate of single DVIU was nearly 8% and significantly decreased with repeated urethrotomies (3). Because of this low success rate, several manipulations have been defined to prevent stricture recurrence such as indwelling Foley catheter, home self-catheterization, and urethral stents. Unfortunately, repeated instrumentation may exacerbate scar formation and complicate subsequent reconstruction and may lead to several complications (1, 4).

Excessive collagen synthesis and changes in composition of the extracellular matrix are the key events in the pathogenesis of urethral stricture. Several previous studies had evaluated the effect of antifibrotic drugs on urethral strictures, such as halofuginone, mitomycin C, botulinum toxin A, somatostatin analog, and glucocorticoids (5-9). Corticosteroid-based drugs were reported to decrease collagen production, and many studies have shown their efficacy on treatment of urethral stricture (10). Mitomycin C (MMC) is also a potent agent that exerts chemotherapeutic and antibiotic activity by inhibiting DNA synthesis. It inhibits mitosis, fibroblast proliferation, protein and collagen synthesis and angiogenesis. This agent plays an important role in tissue healing and scar formation by reducing the release of matrix proteins via the inhibition of proliferative fibroblasts (11).

Clinical studies have shown that MMC has the potential to prevent urethral stricture (6). Both triamcinolone and MMC have anti-proliferative and anti-scarring properties and can be suitable candidates for the treatment of urethral stricture (6, 11, 12). In our study, we compared the efficacy of triamcinolone and MMC on urethral stricture formation and their effect on the inhibition of urethral fibrosis.

## MATERIALS AND METHODS

This study was performed at Namik Kemal University Experimental Animals, Application and Research Center with the approval of Ethics Board for Animal Studies of Namik Kemal University. Twenty-four healthy New Zealand white male rabbits (weight, 2.5-3.5kg) were used. Animals were housed in a temperature-controlled (22±1°C), humidity-controlled, (40%-70%), and light-period controlled (12h/12h light/dark cycle) environment. They were fed with a standard rabbit pellet diet and had access to tap water ad libitum. Before the interventional procedures, ketamine HCl at a dose of 15mg/kg and xylazine at a dose of 6mg/kg were administered intramuscularly for general anesthesia. Rabbit urethras were traumatized as described by Faydaci et al. (13). Briefly, the animals were placed in a supine position, and their genitalia were scrubbed with povidone-iodine solution. An 11F pediatric resectoscope was used for the endoscopic operation. A 2 to 3 mm wide resection on anterior urethra at 5 to 7 o'clock position, 10mm proximal to the external meatus, was performed with pediatric resectoscope using electric energy by the same surgeon (O.K.). The resection was deepened enough to uncover the periurethral tissue to allow urine leakage from the lumen. The urine was deliberately not diverted. No antibiotics were administered.

Animals were randomly divided into three groups, 8 rabbits of each. In group 1 (control), the urethra was traumatized and irrigated with 10mL saline without medical treatment. In group 2, the urethra was traumatized and a cotton wool soaked with 0.5mg/mL MMC was applied to the traumatized area for 5 minutes, and then the urethra was irrigated with 10mL saline. In group 3, the urethra was traumatized and 40mg triamcinolone was injected to the traumatized area as 1mL of injection. At the end of 28 days of surgical manipulation, urethral gross morphology was evaluated by retrograde urethrogram and video-urethroscopy. Contrast medium (20mL of 760g/L meglumine amine diluted by 20mL of 9g/l sodium chloride) was injected slowly and through the urethra by the same researcher, under X-ray vision to visualize the configuration of urethral lumen. The urethral caliber was measured as described by Jaidane et al. (14). For estimation of the percentage of urethral stricture, urethrograms and video-urethroscopy were used. The rate of stricture was defined as the division of the diameter in the narrowest part of stricture to normal diameter of urethra just distal to stricture. Strictures were considered as significant if the urethral lumen diameter decreased more than 50%. The rabbits were euthanized with high dose of pentothal, and whole urethra was removed for histopathologic evaluation.

### 

#### Histologic examination

The urethra specimens were individually immersed in Bouin's solution, dehydrated in alcohol and embedded in paraffin. 5μm thick sections were obtained and subjected to hematoxylin and eosin and Masson trichrome staining to assess fibrosis, epithelium, and collagen density. The urethra tissues were examined and evaluated in random order under blindfold conditions with standard light microscopy by a histologist. The Masson's trichrome staining method was used to investigate fibrotic degree. A score of 0 to 3 was assigned as follows based on the degree of staining and fibrosis: Negative, absence of staining and fibrosis (0 points); mildly positive, slight staining and fibrosis (<25%, 1 point); moderately positive, moderate staining and fibrosis (25%-50%, 2 points); and strongly positive, strong staining and severe fibrosis (>50%, 3 points). Progression from strongly positive to negative was considered to be significant (15). Sections were photographed using a Nikon 50i photomicroscope and NIS elementary software.

#### TUNEL assay

Apoptosis was evaluated by the terminal dUTP nick end-labeling (TUNEL) assay. The TUNEL method, which detects fragmentation of DNA in the nucleus during apoptotic cell death in situ, was employed using an apoptosis detection kit (ApopTag^®^ Peroxidase In Situ Apoptosis Detection Kit, Cat. No. S7100, Millipore, USA). The number of TUNEL-positive cell was evaluated semi-quantitatively; 0, no positive cells; 1, less than 10% positive cells; 2, 10-50%; and 3, >50%.

### Statistical analysis

All data were analyzed with the Statistical Package for the Social Sciences for Windows software (Version 17.0 SPSS, Chicago, IL). Data were presented as mean and standard deviation or percentage. Data in independent groups were analyzed for normalcy with Kolmogorov-Smirnov test and further evaluated with independent t-test or Mann-Whitney U test. Data in dependent groups were analyzed with paired t-test or Wilcoxon signed test after evaluation of normalcy with Kolmogorov-Smirnov test.

## RESULTS

**Figure 1:**
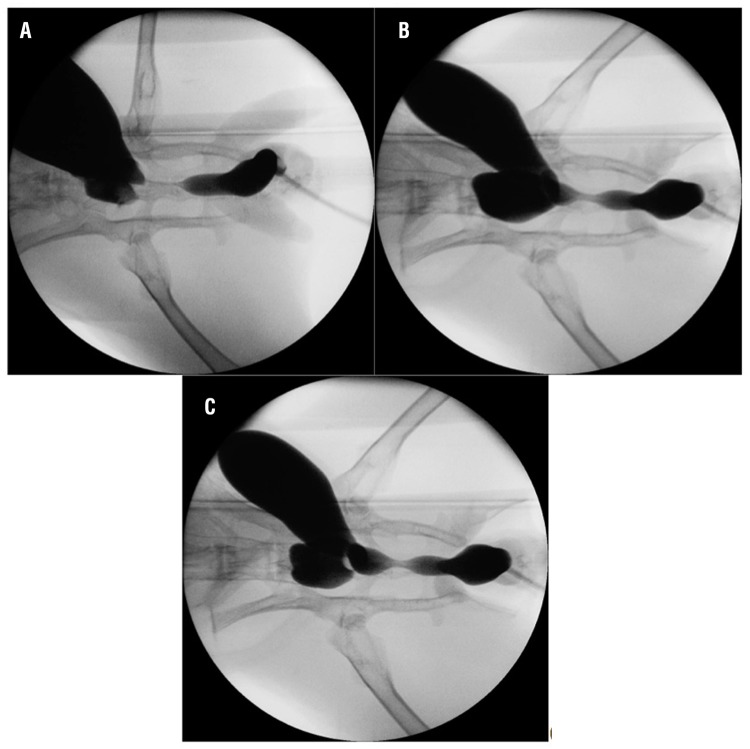
Urethral stricture in groups on retrograde uretrography on postoperative 28^th^ day. (a) Control group. (b) Mitomycine-C group. (c) Triamcinolone group.

**Figure 2:**
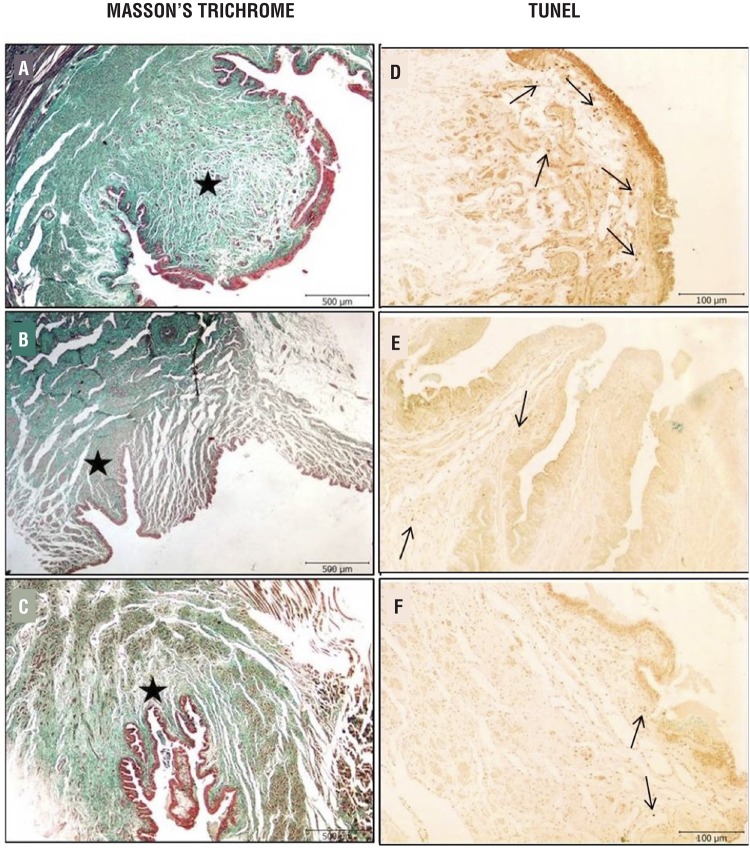
Representative urethra tissue photographs of Masson's trichrome and TUNEL staining. Masson's trichrome; **(a)** Urethral stricture group animals showing extensive collagen deposition are recognized as green in the submucosal connective tissue **(b)** Mitomycin-C treated rats significant less collagen deposition in the urethra **(c)** Triamcinolone treated rats significantly less collagen deposition in the urethra. **Asteriks:** collagen fibers (Masson's trichrome, scale bar: 500μm). TUNEL; The number of apoptotic cells in the submucosal connective tissue were quantitatively higher in urethral stricture groups than control groups **(d, e)**. Treatment of mitomycin markedly reduced the number of apoptotic cells **(f)**. **arrow:** TUNEL positive cells. (TUNEL staining, scale bar: 100μm).

All rabbits survived during the study and voided spontaneously after the procedure. We encountered no major clinical complication. There were statistically significant differences in urethral diameters and in lumen reduction rate, between the control and study groups (Table-1). Both MMC and triamcinolone groups had significantly lower rate of stricture compared to control group (P<0.001). On the other hand, there was no difference between treatment groups in terms of urethral diameters and lumen reduction rate (Table-1), (Figure-1). Light microscopic examination in the control group showed an extensive collagen deposition in the mucosal connective tissue and increased fibroblasts (Figure-2A). Compared to control group, all treatment groups (particularly MMC) exhibited mild fibrosis, less collagen bundle irregularity, and lower numbers of fibroblasts (Figures 2B and 2C) (p=0.003).

**Table 1:** Urethral diameter, lumen reduction rate of rabbits in different treated groups.

	Urethral diameter (mm, mean±SD)	Lumen reduction (%) (mean ± SD)	Fibrosis score (Masson) (mean± SD)	TUNEL score (mean±SD)
Control	2,67 ± 0.49	0,91 ± 0.04	2.75 ± 0.46	2,37 ± 0.51
Mitomycin-C	6,85 ± 0.49	0,45 ± 0.13	1.50 ± 0.53	1.50 ± 0.53
Triamcinolone	6,49 ± 0.57	0,49 ± 0.14	2.00 ± 0.53	1.75 ± 0.46
	**(a)** **p= <0.001**	**(a)** **p= <0.001**		
(p) Value	**(b)** **p= <0.001**	**(b)** **p= <0.001**	p=0.003	p=0.034
	(c)p = 0.377	(c)p = 0.273		

(a) Statistical analysis between control group and Mitomycin-C group;

(b) Statistical analysis between control group and Triamcinolone group;

(c) Statistical analysis between Mitomycin-C group and Triamcinolone group

The Tunnel assay showed that the number of apoptotic cells in the submucosal connective tissue was quantitatively higher in control groups than the treatment groups (Figures 2D, 2E and 2F). Treatment with triamcinolone and especially MMC markedly reduced the number of apoptotic cells (p=0.034) (Table-1).

## DISCUSSION

Urethral stricture is a serious disease that causes voiding dysfunction, which adversely affects quality of life and can trigger a chain of events that can lead to renal failure (16). Most of the preferred treatment techniques like DVIU has low success rates for the treatment of urethral stricture. Open surgery is much more effective with long-term cure rates of 90-95%. On the other hand, open surgery is a complicated technique and requires expertise with significant complications (3).

Although the exact pathophysiology of urethral stricture remains unknown, fibrosis caused by excessive collagen synthesis and changes in the composition of the extracellular matrix have been suggested as pathophysiological mechanisms (5, 12). Any drug or procedure that can delay fibrosis and prevent stricture recurrence after internal urethrotomy would ultimately result in an increase of surgical success rates, patient comfort and would decrease treatment costs. Therefore, studies have been performed to explore different molecules for preventing fibrosis and urethral stricture recurrence (5-9, 16, 17).

Halofuginone is a specific inhibitor of collagen type I synthesis by fibroblasts. Nagler showed that local or oral halofuginone prevented stricture rmation and collagen a1 gene expression, and reduced collagen content (5). In another study rapamycin was shown to be effective in inhibiting fibroblast proliferation and collagen expression (17). Metalloproteinase-1 is another agent that was reported to induce lower collagen concentration in the traumatized region, and it was proposed to be used as an agent to preserve urethral patency (16). All these experimental studies showed that inhibition of fibroblast proliferation and collagen expression is critical for preserving the patency of the urethral lumen. In the present study, we used similar criteria to assess the efficacy of MMC and triamcinolone on preventing urethral stricture.

Mitomycine C is an alkylating antineoplastic antibiotic derived from Streptomyces caespitosus. It inhibits DNA synthesis by cross-linking DNA between adenine and guanine. It is not cell cycle specific and suppresses cellular RNA and protein synthesis. By this way, it delays healing process by preventing replication of fibroblasts and epithelial cells and inhibiting collagen synthesis (18). It was shown that MMC improved the success rates of myringotomy and trabeculectomy by preventing fibroblast proliferation and development of fibrosis (19, 20).

Mazdak et al. injected MMC into the urethral submucosa and reported that patients with MMC injection had lower rates of stricture recurrence (6). Opposing this study, some researchers proposed that submucosal injection could increase the complication rate and reduce the duration of the effective dose within the tissue, which yielded a scientific discussion (21). Ayyildiz et al. assessed the efficacy of MMC for preventing urethral scar by applying the agent topically to the traumatized region in rats (22). They concluded that locally applied MMC significantly reduced fibrosis in a dose-independent manner. We also applied MMC topically, and found that it was effective for reducing urethral stricture rate. Urethral diameter was 6.85 (5.90-7.55) and the stricture ratio was 0.45 (0.33-0.59). These values were statistically significant when compared with the control group.

Triamcinolone reduces fibrosis formation by inhibiting collagen synthesis. It increases collagenase production, and lowers the levels of collagenase inhibitors (23). Corticosteroids are extensively used in treating mucosal strictures and skin scars (10, 24). There is a very limited number of studies reporting its efficacy in treating urethral strictures (25, 26). In the present study triamcinolone significantly reduced stricture formation which corroborates with previous studies.

We also compared the efficacy of MMC and triamcinolone to state whether one of them are superior to other in preventing urethral stricture. Although our results demonstrated that differences between MMC and triamcinolone groups were not statistically significant, MMC was slightly superior on preventing urethral stricture. Both agents were statistically superior on preventing urethral stricture compared to control group. Hematoxylin-eosin staining results confirmed that there was less fibrosis and lower collagen content in the two groups treated with MMC and triamcinolone. Also Tunnel assay results showed that the number of apoptotic cells in the submucosal connective tissue was lower in MMC and triamcinolone groups compared to control group.

The considerably short follow-up period can be considered as a study limitation. Many strictures may recur within 2 year following internal urethrotomy. Pansadoro et al. suggested that a minimum follow-up of 5 years is required to assess the results of treatment of urethral stricture (27). On the other hand, the literature fully supports our local data, and despite the limitations, our results may provide useful insight for clinicians who want to offer additional medical therapy in order to prevent urethral stricture recurrence for patient that has undergone internal urethrotomy.

## CONCLUSIONS

In the view of efficacy and safety, MMC and triamcinolone hve the potential to replace the use of stents, clean intermittent catheterization, or long term catheters following internal urethrotomy. There were no statistically significant differences between two agents in terms of preventing US formation in the present study. Future studies may provide more detailed information for the usage of these drugs in urethral stricture.
